# Recent Insights Into the Molecular Mechanism of Toll-Like Receptor Response to Dengue Virus Infection

**DOI:** 10.3389/fmicb.2021.744233

**Published:** 2021-09-16

**Authors:** Mohammad Enamul Hoque Kayesh, Michinori Kohara, Kyoko Tsukiyama-Kohara

**Affiliations:** ^1^Transboundary Animal Diseases Centre, Joint Faculty of Veterinary Medicine, Kagoshima University, Kagoshima, Japan; ^2^Department of Microbiology and Public Health, Faculty of Animal Science and Veterinary Medicine, Patuakhali Science and Technology University, Barishal, Bangladesh; ^3^Department of Microbiology and Cell Biology, Tokyo Metropolitan Institute of Medical Science, Tokyo, Japan

**Keywords:** innate immune response, Toll-like receptors, dengue virus, infection, vaccine

## Abstract

Dengue is the most prevalent and rapidly spreading mosquito-borne viral disease caused by dengue virus (DENV). Recently, DENV has been affecting humans within an expanding geographic range due to the warming of the earth. Innate immune responses play a significant role in antiviral defense, and Toll-like receptors (TLRs) are key regulators of innate immunity. Therefore, a detailed understanding of TLR and DENV interactions is important for devising therapeutic and preventive strategies. Several studies have indicated the ability of DENV to modulate the TLR signaling pathway and host immune response. Vaccination is considered one of the most successful medical interventions for preventing viral infections. However, only a partially protective dengue vaccine, the first licensed dengue vaccine CYD-TDV, is available in some dengue-endemic countries to protect against DENV infection. Therefore, the development of a fully protective, durable, and safe DENV vaccine is a priority for global health. Here, we demonstrate the progress made in our understanding of the host response to DENV infection, with a particular focus on TLR response and how DENV avoids the response toward establishing infection. We also discuss dengue vaccine candidates in late-stage development and the issues that must be overcome to enable their success.

## Introduction

Dengue is the most important arthropod-borne human viral infection caused by dengue virus (DENV); it is a global health concern in many tropical and subtropical countries and areas, and most outbreaks occur in urban and semi-urban areas (Guzman et al., 2010; World Health Organization (WHO), 2020). *Aedes aegypti* is the primary vector for DENV transmission, while *Aedes albopictus* is a less common vector (Lambrechts et al., 2010). DENV is a positive-sense, single-stranded RNA virus with a genome of 10.7 kb under the family Flaviviridae and the genus Flavivirus (Kuhn et al., 2002). The DENV genome encodes three structural proteins, the capsid (C), membrane (M), and envelope (E), and seven non-structural proteins (NS1, NS2A, NS2B, NS3, NS4A, NS4B, and NS5) (Kuhn et al., 2002; Guzman et al., 2010). DENV has four genetically and antigenically distinct serotypes: DENV-1, DENV-2, DENV-3, and DENV-4 (Simmons et al., 2012). While protection against homologous reinfection is lifelong, only short-term protection can be provided against heterologous infection (Gibbons et al., 2007; Aguas et al., 2019). Moreover, heterologous infection may cause severe dengue, possibly because of the antibody-dependent enhancement (ADE) effect (Dejnirattisai et al., 2010; Katzelnick et al., 2017). DENV infection causes a spectrum of illness in humans, ranging from asymptomatic to mild fever, as well as potentially life-threatening severe dengue such as dengue hemorrhagic fever (DHF) and dengue shock syndrome (DSS; Harris et al., 2000). Severe dengue is characterized by plasma leakage, hemorrhagic tendencies, organ failure, shock, and death (Srikiatkhachorn, 2009). However, the mechanisms underlying dengue-related diseases are not completely understood. It has been reported that innate immunity plays a pivotal role during early DENV infection stages in both priming protection and disease induction (Costa et al., 2013). Type I interferons (IFNs) are important for host defense against viral infections; during infection, viruses are recognized by different pattern recognition receptors (PRRs), including Toll-like receptors (TLRs), retinoic acid-inducible gene I (RIG-I)-like receptors (RLRs), and nucleotide-binding oligomerization domain-like receptors [NOD-like receptors (NLRs)]. This recognition may lead to the induction of the genes encoding type I IFNs through several distinct signaling pathways (Kawai and Akira, 2011; McNab et al., 2015).

According to a recent estimate, approximately 390 million DENV infections occur annually worldwide, and 3.9 billion people are at risk of acquiring infections (Bhatt et al., 2013). During the last two decades, there has been a significant increase in the incidence of dengue, which has risen from 505,430 cases in 2000 to over 2,400,138 and 3,312,040 cases in 2010 and 2015, respectively (World Health Organization (WHO), 2020). The number of deaths also increased from 960 to over 4,032 between 2000 and 2015, emphasizing the urgent need for a safe and effective dengue vaccine (World Health Organization (WHO), 2020). The global rise in dengue is influenced by several factors, including climate change, population growth, high population density, unplanned rapid urbanization and construction, absence of reliable piped water, and ineffective vector control strategies (Wilder-Smith et al., 2010; Simmons et al., 2012; Struchiner et al., 2015). Moreover, the rapid global spread of dengue is associated with human mobility through air travel (Tian et al., 2017). Vector control strategies should assist with controlling dengue infection (Kittayapong et al., 2017; Ritchie, 2018); however, vaccines may provide the best intervention from the perspectives of both public health and economic concerns (Wilder-Smith, 2020). The first dengue vaccine, chimeric yellow fever 17D-tetravalent dengue vaccine (CYD-TDV)/Dengvaxia, was developed by Sanofi Pasteur Co., and licensed in 2015 (Hadinegoro et al., 2015). This vaccine is now only recommended for use in seropositive individuals. However, there are currently no effective prophylactic and/or therapeutic pan-serotype DENV vaccines (Tremblay et al., 2019). Vaccines that can provide long-term protection against each of the four DENV serotypes by inducing neutralizing antibodies (nAbs) are essential for controlling the disease and the avoidance of ADE-mediated severe dengue (Murphy and Whitehead, 2011). Here, we discuss the current progress in our understanding of the host innate immune response to DENV infection, particularly the TLR response and its evasion/inhibition by DENV to establish infection. We also discuss dengue vaccine candidates in the late stages of development, highlighting the challenges that must be overcome regarding these candidate vaccines.

## Innate Immune Response to DENV Infection

A complex series of events are involved in the interactions between a virus and the host immune system that determine the outcome of an infection (Kayesh et al., 2019). The innate immune response is a key component of the host defense system and acts as the first line of immune defense against many viral infections (Zuniga et al., 2015). However, the innate immune response may not always be protective; it may also contribute to pathology, particularly when the response is uncontrolled (Modhiran et al., 2015). TLR signaling is involved in the regulation of both pro- and anti-inflammatory cytokines, linking early innate immune responses and adaptive immunity (Ozato et al., 2002). Moreover, cytokines show multifaceted interactions and regulate immune responses, which may induce disease pathogenesis (Sanapala and Pola, 2020). Notably, TLR activation may act as a double-edged sword, and it is possible to enhance immune-mediated pathologies instead of inducing an immune response to protect against pathogens (Salaun et al., 2007; Huang et al., 2008; Yokota et al., 2010). Therefore, a complete understanding of the innate immune response induced by DENV infection is essential for understanding DENV pathogenesis and its effective control and prophylactic measures. Here, we discuss the innate immune response against DENV infection, with a particular focus on TLR response. For further information on the innate immune response to DENV infection, there are some previously published reviews that could provide more wide information (Navarro-Sanchez et al., 2005; Mathew, 2018; Rathore and St John, 2018; Uno and Ross, 2018; King et al., 2020; Malavige et al., 2020).

## TLR Response to DENV Infection

The pathogenesis of dengue is complex, and its underlying mechanisms are not fully understood. The clinical outcome of DENV infection depends on the complex interplay between the virus and host immune response (Costa et al., 2013; Yacoub et al., 2013). TLRs play a crucial role in innate immunity against viral infections and can activate NF-κB, a critical transcriptional factor (Rahman and McFadden, 2011). A previous study showed that FcγRI and FcγRIIa synergistically facilitate the entry of DENV antibody complexes into THP-1 (human monocytic cell line) cells, and an interplay between DENV and pre-existing antibodies from previous DENV infection may subvert the innate immune response by downregulating TLR signaling (Modhiran et al., 2010). In a previous study, a differentially expressed TLR profile was reported in children with severe dengue, wherein increased expression of TLR7 and TLR4 transcript variant 3 (TLR4R3) and decreased expression of TLR1, TLR2, TLR4R4, and TLR4 cofactor CD14 were observed (de Kruif et al., 2008). As dendritic cells (DCs), monocytes are considered important target cells for DENV infection both *in vitro* and *in vivo* (Halstead et al., 1980; Wu et al., 2000). A previous study reported a reduced number of CD14(+) human leukocyte antigen (HLA)-DR (+) monocytes in patients with severe dengue compared to those with mild dengue and controls (Azeredo et al., 2010). In addition, CD14(+) monocytes expressing TLR2 and TLR4 were increased in peripheral blood from mild dengue patients compared to in that of patients with severe dengue, suggesting the protective role of TLR2 and TLR4 in this setting (Azeredo et al., 2010). Increased TLR3 and TLR9 expression was found in DCs of patients with dengue fever (DF) early in infection, and poor stimulation of TLR3 and TLR9 was observed in DCs from patients with severe manifestations, suggesting a role for TLRs in dengue pathogenesis (Torres et al., 2013). Additionally, lower TLR2 expression was found in patients with DF compared to in those with DHF (Torres et al., 2013). A previous study reported the involvement of TLR3, 7, and 8 in the recognition of the DENV-2 NGC strain, and a strong induction of IL-8 and IFN-α/β responses mainly produced by TLR3 signaling was found to inhibit viral replication in HEK293 and U937 cell lines (Tsai et al., 2009). However, the DENV-2 NGC strain induced IL-6 expression in U937 cells, but not in THP-1 cells. This finding is in contrast with a previous study that reported IL-6 expression in THP-1 cells by DENV-2 16681 strain infection (Chareonsirisuthigul et al., 2007); however, this discrepancy could be attributed to the difference between the DENV-2 strains. A previous study showed that TLR3 can inhibit the replication of DENV-2 in HepG2 cells by inducing IFN-β expression (Liang et al., 2011). Another study reported that TLR3, 7, and 8 can induce inflammatory and humoral responses in rhesus macaques and suppress DENV-1 Western Pacific 74 strain replication (Sariol et al., 2011). Based on myeloid and plasmacytoid dendritic cells (mDCs and pDCs), DENV replication and cytokine responses may differ. TLR7-mediated recognition of DENV-2 (strain 16803) and increased IFN-α production in pDCs has been previously reported (Sun et al., 2009). Nasirudeen et al. (2011) showed that DENV infection induces intracellular RNA virus sensors, including RIG-I, MDA5, and TLR3, and essential components of host defense. In addition, RIG-I and MDA5 showed an inhibitory role against DENV-1 in HuH-7 cells alongside significantly increased IFN-β expression (Nasirudeen et al., 2011). However, despite the knockdown of RIG-I and MDA5, the level of IFN-β production was increased upon DENV-1 infection due to TLR3 activation (Nasirudeen et al., 2011). TLR3 can detect double-stranded RNA (dsRNA), a molecular pattern associated with viral infection (Alexopoulou et al., 2001). Moreover, in a recent study, poly(ADP-ribose) polymerase 9 (PARP9), a non-canonical sensor for RNA virus was shown to recognize and bind viral or poly I:C dsRNA activating the phosphoinositide 3-kinase (PI3K) and AKT3 pathway to produce IFN-α, independent of mitochondrial antiviral signaling (MAVS) and exerts antiviral effects (Xing et al., 2021a). However, the role of PARP9 in DENV infection remains to be identified. In addition, a recent study demonstrates that RNA helicase DEAH-box helicase 15 (DHX15) exerts its antiviral role independent of RIG-I and MDA5 by inducing the production of IFN-β, IFN-λ3, and IL-18 in response to dsRNA poly I:C or enteric RNA virus rotavirus or reovirus infection (Xing et al., 2021b), which highlights the importance of future investigation of the antiviral role of DHX15 in DENV infection. However, another study showed the induction of TLR2 and TLR6 signaling pathways and downstream IL-6 and TNF-α production by DENV NS1 protein after DENV-2 infection in human peripheral blood mononuclear cells (PBMCs; Chen et al., 2015). Notably, DENV-2-infected and NS1 protein-treated TLR6-/- mice showed higher survivability than DENV-2-infected and NS1 protein-treated wild-type mice, suggesting a role for TLR6 in DENV immunopathogenesis in this model (Chen et al., 2015). Modhiran et al. also reported that NS1 activates TLR4 signaling pathways, leading to the production of proinflammatory cytokines and chemokines (Modhiran et al., 2015, 2017). However, a previous study reported that DENV NS1 does not inhibit TLR3 signaling (Baronti et al., 2010). Sirtuins (SIRTs 1–7) are a family of nicotinamide adenine dinucleotide (NAD)-dependent deacetylases that play an important role in controlling inflammation by regulating immune gene transcription. In a recent study, Li et al. (2018) demonstrated that SIRT6 negatively regulates the DENV-induced inflammatory response through TLR3 and RLR signaling pathways. NS1-induced platelet activation *via* TLR4 on platelets has been reported, which may lead to thrombocytopenia and hemorrhage (Chao et al., 2019; Quirino-Teixeira et al., 2020). It has been reported that individuals with a heterozygous genotype for TLR4 Asp299Gly and Thr399Ile polymorphisms showed higher susceptibility to DENV infection (Sharma et al., 2016), suggesting the role of TLR4 in DENV infection. A recent study reported decreased expression of TLR3, 7, and 9 in monocyte-derived DCs (MDDCs) after oral supplementation with vitamin D (4,000 IU/day) (Martinez-Moreno et al., 2020). In addition, a decrease in IL-12 and IL-8 levels and an increase in IL-10 expression were observed, and the cells showed reduced susceptibility to DENV-2 infection (Martinez-Moreno et al., 2020). However, a previous study reported no significant change in TLR expression after oral supplementation with vitamin D (Giraldo et al., 2018), which could be attributed to differences between cell types.

**FIGURE 1:**
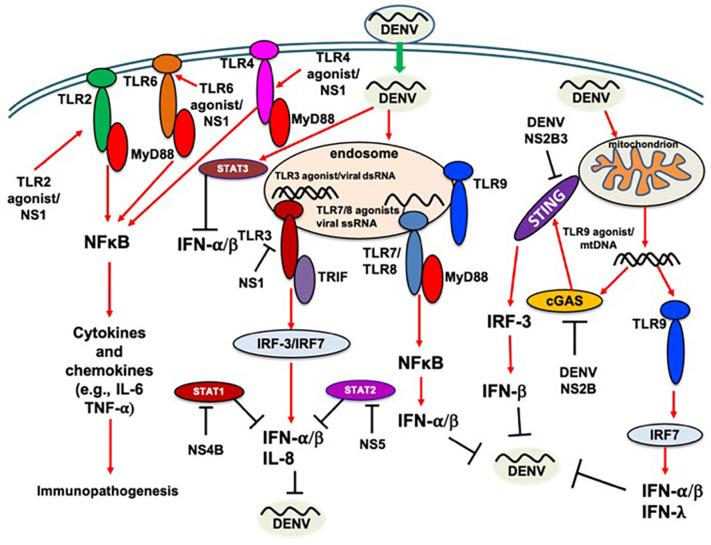
TLR response to DENV infection. Red arrow indicates the induction/activation of components of TLR signaling by DENV; black line indicates the inhibition or cleavage or degradation of the host immune components/response or inhibition of DENV replication, as appropriate.

It has been demonstrated that DENV NS1 protein alone may induce vascular leakage and inflammatory cytokine secretion. This could be prevented using NS1-immune polyclonal mouse serum or monoclonal antibodies against NS1, suggesting that DENV NS1 is a key player in DENV-induced immunopathogenesis (Beatty et al., 2015). TLR9 knockdown was reported to inhibit DENV-induced IFN-λ1, IFN-λ2, IFN-λ3, and IFN-β1 mRNA expression, suggesting the antiviral role of TLR9 signaling in DENV infection (Lai et al., 2018). A previous *in vitro* study reported the potential of IFN-λ1 to inhibit DENV-2 replication (Palma-Ocampo et al., 2015), the production of which may involve TLR3, IRF-3, and NF-κB molecules; furthermore, NS1 protein may remain the main viral component that induces IFN-λ1 production (Hsu et al., 2016). It was recently demonstrated that TLR2, together with its coreceptors CD14 and TLR6, has a potential role in modulating vascular integrity during DENV infection (Aguilar-Briseno et al., 2020). Analysis of TLR expression in DENV-infected corneas revealed upregulation of TLR4, TLR7, TLR9, and TLR10 (Parthasarathy et al., 2018), which may lead to an increased innate proinflammatory response in the cornea. In a recent study of DENV and chikungunya virus (CHIKV) coinfection, the TLR7 and TLR8 polymorphisms have been linked to susceptibility to or protection against infections (Sengupta et al., 2021), suggesting the crucial role of TLRs in DENV infection. In our previous study, we showed that tupaia lung fibroblast cells are susceptible to all four serotypes of DENV infection, and tupaia TLR8 may possess antiviral potential against DENV infection (Kayesh et al., 2017). The pathogenesis of dengue is immune-mediated and complex, and regulatory T cells (Tregs) may suppress the immune response and contribute to better prognosis. A recent study reported altered profiles of Tregs and associated cytokines in mild and moderate dengue. Mild cases had significantly higher levels of Tregs, and IL-6 and IL-8 levels were found to be negatively correlated with Treg levels (Tillu et al., 2016). However, the molecular pathways involved in Treg proliferation are poorly understood. A double-faced implication of CD4 + Foxp3 + Tregs expanded by acute dengue infection *via* the TLR2/MyD88 pathway has been reported in a murine model (George et al., 2020). DENV infection has been reported to induce CD4 + Foxp3 + Treg proliferation *via* the TLR2/MyD88 pathway. Furthermore, dengue-infected hosts are more susceptible to sepsis, which could be due to early TLR2-dependent proinflammatory cytokine production (George et al., 2020). CD4 + Foxp3 + Treg cells may dampen induction of the immune response; therefore, these cells should be contained to allow effective protection against pathogens, including viruses. Based on these findings, it is important to investigate the implications of high CD4 + Foxp3 + Treg levels in DENV infections. Aside from RNA sensors, DENV can activate cytosolic DNA-specific sensors and cGAS signaling through the release of mitochondrial DNA (mtDNA) into the cytosol. DENV can also trigger a cGAS-mediated antiviral response, which highlights an indirect activation of DNA-specific innate immune signaling pathway by DENV infection (Sun et al., 2017). Moreover, the contributions of DENV-induced immune activation by TLR9 and cGAS are comparable (Sun et al., 2017; Lai et al., 2018). For simplicity, the above findings obtained in different cells/systems have been indicated in Figure 1 without indication of cell type/system, and highlighting that various TLRs are implicated in DNEV replication (Figure 1), which may impact viral pathogenesis. Therefore, a complete understanding of TLRs in DENV infection is critical for designing a successful therapeutic or preventive intervention.

## Inhibition of Innate Immune Response by DENV

An improved understanding of DENV immune evasion is crucial for the rational development of anti-DENV tools. Like many other viruses, DENV employs a variety of mechanisms to avoid, reduce, or disrupt antiviral immunity and establish infection. Mice with impaired type I IFN signaling have enhanced susceptibility to DENV infection, suggesting the role of IFN in inhibiting DENV infection (Zust et al., 2014). Inhibition of type I IFN signaling is a crucial mechanism of DENV immune evasion. TLRs play a role in the antiviral innate immune response, as indicated above, and DENV also possesses mechanisms to antagonize the host antiviral innate immune response to establish infection. Here, we discuss how DENV evades the host innate immune response to establish infection, with a particular focus on the inhibition of TLR signaling (Figure 1) and type I IFN production. It has been shown that DENV cannot infect IFN-α/β-treated human cells; however, IFN treatment after DENV infection could not inhibit viral replication, suggesting that DENV possesses the tools to inhibit IFN (Diamond et al., 2000). In a previous study, DENV-2 proteins NS2A, NS4A, and NS4B were identified as IFN antagonists (Munoz-Jordan et al., 2003). It has been shown that NS4B blocks the IFN-induced signal transduction cascade by interfering with signal transducer and activator of transcription (STAT) 1 activation (Munoz-Jordan et al., 2003). It has been reported that the catalytically active NS2B3 protein complex antagonizes type I IFN response in human DCs (Rodriguez-Madoz et al., 2010a). Furthermore, DENV infection in primary human DCs reportedly does not induce IRF-3 phosphorylation, which results in the inhibition of type I IFN synthesis (Rodriguez-Madoz et al., 2010b). However, non-canonical IRF-3, IRF-5, and IRF-7-independent antiviral defense mechanisms in DENV infection have been demonstrated in AG129 mice, which are mediated by IRF-1 through IL-12/IFN-γ production (Carlin et al., 2017). A previous study also showed the induction of IRF-1 and IRF-7 in DENV-2 infection in A549 cells (Chang et al., 2006); however, the mechanism of IRF-1 induction remains to be confirmed. In a previous study, Jones et al. (2005) reported that DENV can subvert the human IFN response by downregulating STAT2 expression. Several studies have reported that DENV NS5 is a potent antagonist of type I IFNs. These type I IFNs can degrade STAT2, a necessary component of the type I IFN signaling pathway, and thus help in DENV replication (Mazzon et al., 2009; Ashour et al., 2010; Morrison et al., 2013). DENV NS1 protein levels in serum may induce changes in innate immune parameters, which may contribute to different clinical outcomes of DENV infection. Carvalho et al. (2014) showed that higher NS1 levels in serum may induce a reduced TLR4 response to LPS. Recently, Murphy Schafer et al. (2020) introduced an additional mechanism of TLR signaling disruption by DENV-2. DENV-2 infection induces SIAH1 expression, resulting in SIAH1 binding and ubiquitination of MyD88, an adaptor protein of TLR signaling, thereby dampening the host innate immune response and promoting viral replication.

Although originally stimulator of interferon genes (STING) was described as a response to cytosolic DNA sensing (Zhang et al., 2011), a recent study indicated that STING is also activated upon RNA virus infection (Holm et al., 2016). STING plays an important role in the induction of type I IFN responses against viral infections (Ishikawa et al., 2009). DENV can inhibit IFN production by targeting the cGAS/STING signaling pathway, and it has been shown that the DENV NS2B3 protease complex can cleave human STING, but not mouse STING (Aguirre et al., 2012; Yu et al., 2012; Stabell et al., 2018). Stabell et al. (2018) also discovered that DENV cannot inactivate STING in most primates, including chimpanzees (*Pan troglodytes*), rhesus macaque (*Macaca mulatta*), and common marmosets (*Callithrix jacchus*). A recent study showed that DENV NS2B can target cGAS for degradation, thus preventing mtDNA sensing through cGAS during DENV infection (Aguirre et al., 2017). Another study showed that DENV infection inhibits STING-mediated innate immunity in a haplotype-specific manner (Su et al., 2020). Moreover, in a murine model, it has been shown that DNEV-induced illness may result from dysregulated STING-mediated vasculopathy (Warner et al., 2017). In a recent study, Ye et al. (2021) showed that USP18 induced by DENV-2 infection is a critical host factor used by DENV-2 to antagonize IFN-α production. Ye et al. (2021) demonstrated that DENV-2 infection increased USP18 expression; USP18 overexpression enhanced DENV-2 replication, while USP18 silencing inhibited DENV-2 replication by activating the IFN-α-mediated JAK/STAT signaling pathway. A recent study reported a dose-dependent inhibition of DENV replication with (E)-guggulsterone, which stimulates nuclear factor erythroid 2-related factor 2 (Nrf2)-mediated heme oxygenase-1 (HO-1) expression; this expression increased the antiviral IFN response and downstream antiviral gene expression by blocking DENV NS2B/3B protease activity (Chen et al., 2021). However, in an *in vitro* study, Ferrari et al. (2020) showed that the DENV NS2B3 protease complex can target and promote Nrf2 degradation. A recent study revealed that DENV could utilize STAT3 as a proviral factor for its propagation in A549 cells. In these cells, DENV strategically tweaks the negative regulator of type I IFN signaling, STAT3, to evade host type I and type III IFN responses by upregulating STAT3 expression and activation (Srivastava et al., 2021).

## DENV Vaccines Under Development

The lack of an effective vaccine that can simultaneously protect against the four DENV serotypes in naive individuals remains an unsolved issue. Moreover, the use of the CYD-TDV vaccine as a primary prevention strategy for DENV infection is compromised. However, the limited efficacy and safety issues of the CYD-TDV vaccine have led to the development of safer and more effective vaccine candidates for dengue prevention and control. Five types of dengue vaccines are currently under development: a live-attenuated vaccine, an inactivated virus vaccine, a subunit vaccine, a viral vectored vaccine, and a DNA vaccine (Deng et al., 2020; Huang et al., 2021). For details of the dengue vaccine candidates and their development, please refer to the study by Pinheiro-Michelsen et al. (2020). Several vaccine candidates, including TAK-003, TV003/TV005, TDENF17/F19, TDEN PIV, V180, and TVDV, are present in the clinical pipeline (Idris et al., 2021). Here, we will briefly discuss the first dengue vaccine, CYD-TDV, and other dengue vaccine candidates, including TAK-003 and TV003/TV005, in their late-stage development. The advances in these vaccines are summarized in Table 1.

**TABLE 1:** Summary of DENV vaccines in late-stage development.

**Vaccine name**	**Vaccine type**	**Developer/sponsor/manufacturer**	**Vaccine formulation**	**Clinical phase**	**Outcome (strengths)**	**Outcome (limitations)**	**References**
CYD-TDV/Dengvaxia	Live-attenuated tetravalent vaccine	Sanofi Pasteur	Replacing prM/E RNA of YF17D with corresponding sequence of DENV-1 to DENV-4	IV (post-license evaluation)	First licensed dengue vaccine; will act as platform for further refinement of candidate vaccines	Age limit; prevaccination screening required for vaccination and only seropositive individuals are recommended for vaccination; increased risk of severe dengue in seronegative subjects; vaccine efficacy depends on age, serotype, and serostatus; three-dose schedule; long-term safety assessment limits use	Sridhar et al., 2018; Deng et al., 2020; Pinheiro-Michelsen et al., 2020; DiazGranados et al., 2021
TAK-003; DENVax	Live-attenuated tetravalent vaccine	Takeda/Inviragen	DENV2 PDK-53 backbone with prM/E regions from DENV-1, -3, and -4	Phase III	Well tolerated; induces Abs against all four serotypes irrespective of age and prevaccination serostatus	Efficacy varies depending on serotypes; lower protection rates against DENV-3 and DENV-4	Biswal et al., 2020; Lopez-Medina et al., 2020
TV003/TV005	Live-attenuated tetravalent vaccine	NIH (United States); Butantan Institute (Brazil)	rDEN1Δ30, rDEN2/4 Δ30, rDEN3Δ30/31, and rDEN4Δ30 (TV003)	Phase II/III	Well tolerated; balanced immune response in 76% of vaccines; Ab detection in 91.7% of subjects; shows more efficacy against DENV-2 compared to CYD-TDV; effective with administration of a single dose	Adverse reaction (mild rash)	Whitehead et al., 2017; Deng et al., 2020; Durbin, 2020; Nivarthi et al., 2021

### CYD-TDV

The first licensed dengue vaccine, CYD-TDV, is a chimeric dengue-yellow fever virus 17D (YF17D) vaccine that was constructed by replacing the prM/E RNA sequences of YF17D with corresponding sequences of the DENV-1–4 serotypes (Guy et al., 2010). Data from phase III trials revealed that vaccine efficacy varies depending on age, serotype, and serostatus, and a low vaccine efficacy for DENV-1 (50.3%) and DENV-2 (42.3%) has been reported (Villar et al., 2015). Moreover, a higher risk of hospitalization has been observed in children younger than 9 years of age (Sridhar et al., 2018). Threat of ADE has also been reported in seronegative individuals who were sensitized by the vaccine (Halstead, 2017). Despite some limitations of the CYD-TDV vaccine, this vaccine should contribute to the control of DENV infection in seropositive individuals in dengue-endemic areas/countries. In a phase III clinical trial in Columbia, it has been observed that the efficacy of CYD-TDV against symptomatic virologically confirmed dengue (VCD) was 67.5%; CYD-TDV was found to be a useful tool to consider as part of an integrated control strategy against endemic dengue (Reynales et al., 2020). In compliance with recommendations by the WHO, a recent study highlighted the importance of prevaccination screening for detecting previous dengue infection during vaccination with CYD-TDV; this is crucial to provide protection against dengue disease and reduce the risk of dengue hospitalization and severe dengue (DiazGranados et al., 2021). Therefore, it is important to develop cost-effective and reliable diagnostic tools for rapid prevaccination screening (Wilder-Smith et al., 2019), which may enhance the use of CYD-TDV and assist with dengue control in dengue-endemic areas. In a randomized, controlled, phase II, non-inferiority study of CYD-TDV in healthy individuals aged 9–50 years, the one- and two-dose groups were compared to the three-dose group. The two-dose CYD-TDV regimen was revealed as an alternative to the licensed three-dose regimen in seropositive subjects at baseline and aged 9 years and older (Coronel-Martinez et al., 2021). Moreover, in low-resource settings, vaccination with a reduced number of doses may lead to improved vaccine compliance and coverage.

### TAK-003

TAK-003 (previously called DENVax), Takeda’s tetravalent dengue vaccine candidate, is based on a live-attenuated DENV-2 (DEN2-PDK-53). The latter vaccine provides the genetic backbone into which three chimeric viruses containing the prM and E proteins of DENV-1, DENV-3, and DENV-4 are inserted (Osorio et al., 2016). Therefore, there is a difference between the vaccine components of Dengvaxia and TAK-003, as the DENV-2 backbone contains the non-structural proteins. In phase I and II clinical trials, TAK-003 was found to be immunogenic and well tolerated; high titers of nAbs were detected against all four serotypes, irrespective of age and prevaccination dengue exposure (Osorio et al., 2014; George et al., 2015; Rupp et al., 2015; Sirivichayakul et al., 2016; Saez-Llorens et al., 2018; Tricou et al., 2020a,b). A phase II, double-blind, placebo-controlled trial of TAK-003 in children aged 2–17 years living in dengue-endemic countries showed antibody responses against all four serotypes, which persisted for 4 years post-vaccination (Tricou et al., 2020b). A phase II clinical trial with one or two doses of a lyophilized TAK-003 revealed seropositivity after only one dose against the DENV-2 serotype; however, seropositivity to all four serotypes was achieved after two doses (Turner et al., 2020). In a multicenter phase III clinical trial, TAK003 showed an overall vaccine efficacy of 73.3%, regardless of the serostatus before vaccination; this efficiency was measured after 17 months of vaccination with two doses administered three months apart (Biswal et al., 2020). However, similar to Dengvaxia, the efficacy of TAK-003 varies depending on the serotype, and lower protection rates have been shown against DENV-3 (48.9%) and DENV-4 (51.0%) (Biswal et al., 2020; Lopez-Medina et al., 2020). TAK-003 immunization reportedly elicits potent cellular immunity against structural and non-structural proteins of all four DENV serotypes; this immunity is maintained for at least 4 months post-vaccination, with focused reactivity against NS1 and NS3 (Waickman et al., 2019). Notably, a previous study reported live-attenuated tetravalent dengue vaccine (TDV)-induced CD8 + T cells targeting NS1, NS3, and NS5 proteins of attenuated DENV-2 (Chu et al., 2015); however, vaccine-induced CD8 + T cell responses were not reported in the subsequent clinical trials of this candidate vaccine.

### TetraVax-DV-TV003/TV003/TV005/Butantan DV

The live-attenuated tetravalent DENV vaccine candidate TV003/TV005 was developed by the Laboratory of Infectious Diseases at the National Institutes of Health. The vaccine was also licensed by several manufacturers for development, including the Butantan Institute, Brazil, which initiated a Phase III clinical trial (Durbin, 2020). TV003/TV005 consists of rDEN1Δ30, rDEN4Δ30, rDEN3Δ30/31, and rDEN2/4Δ30. rDEN1Δ30 and rDEN4Δ30 contain deletions in the 3′ untranslated region (UTR) of DENV-1 and DENV-4, rDEN3Δ30/31 contains an extra deletion in the 3′ UTR of DENV-3, and rDEN2/4Δ30 is a chimeric virus in which the prM/E sequence of DENV-2 replaced those of the DEN4Δ30 vaccine candidate (Kirkpatrick et al., 2015). However, there is a slight difference between TV003 and TV005 with respect to the dose of the rDEN2/4Δ30 component; TV003 contains 10^3^ PFU rDEN2/4Δ30, while TV005 contains 10^4^ PFU (Durbin, 2020). In a phase I trial, TV003/TV005 was found to be immunogenic and well tolerated, and administration of a single dose induced seroconversion to all four DENV serotypes in 74–92% (TV003) and 90% (TV005) of flavivirus-naive adults (Kirkpatrick et al., 2015; Whitehead, 2016). Notably, both the first and second doses (6 months apart) were well tolerated; however, no significant increase in antibody titers was observed upon administration of the booster dose (Kirkpatrick et al., 2015), suggesting that a single dose of the vaccine is likely to prevent viral infection. It has also been reported that a single dose induces robust tetravalent antibody and cellular T cell responses and is comparable to natural dengue infection (Weiskopf et al., 2015). A previous study reported that TV005 vaccination elicits CD4 + cell responses that are closely mirrored, as observed in a population associated with natural immunity (Angelo et al., 2017). TV003 was also found to be well tolerated in flavivirus-experienced individuals and induced robust nAb titers (Whitehead et al., 2017). In a phase I clinical study in 21 dengue-naive individuals with a single dose of TV003, 76% of the subjects developed serotype-specific nAbs. Following challenge with a partially attenuated DENV-2 strain, all 21 subjects were protected, indicating the induction of immunity by each of the vaccine components (Nivarthi et al., 2021). In another study, TV003 was found to be well tolerated, except for a mild rash as an adverse reaction; a single dose induced seroconversion against all four DENV serotypes in 91.7% of subjects, with 100% seroconversion against DENV-2, DENV-3, and DENV-4 (Kirkpatrick et al., 2016). It was observed that a single dose of TV003 or TV005 in flavivirus-naïve subjects induced a cumulative serologic response in 89.8 and 92.1% of cases, respectively (Durbin, 2020), suggesting that TV005 showed slightly better efficacy than TV003. Overall, TV003/TV005 showed improved efficacy compared with CYD-TDV. In a phase II, randomized, multicenter, double-blind, and controlled clinical trial in adults aged 18–59 years, Butantan-DV and TV003 were evaluated. Both Butantan-DV and TV003 were immunogenic and well tolerated; they induced robust, balanced nAb responses against the four DENV serotypes without any serious adverse reactions. However, rash was observed as the most frequent adverse reaction in the groups vaccinated with either Butantan-DV or TV003 (Kallas et al., 2020).

## Challenges to Overcome in DENV Vaccine Development

To date, there is no vaccine that provides cross-protection against all human DENV serotypes (Scherwitzl et al., 2017). The development of a universal dengue vaccine that is equally protective against all serotypes is a long-sought goal in dengue research. However, it has become challenging to develop a universal DENV vaccine owing to some limitations. One of the major limitations in DENV vaccine development is the existence of multiple DENV subtypes and different virulence mechanisms in different strains (Idris et al., 2021). ADE of disease appears to be a public health concern regarding the development of vaccines and antibody therapies; the mechanisms underlying antibody protection against viruses might have the potential to enhance infection or induce immunopathology (Arvin et al., 2020). DENV vaccine development is also threatened by ADE in heterotypic DENV infection in the presence of subprotective/non-neutralizing antibodies that may cause severe dengue (Katzelnick et al., 2017). Previous DENV vaccine studies revealed human clinical safety risks related to ADE (Dejnirattisai et al., 2010; Halstead, 2017; Sridhar et al., 2018). A recent study indicated that the TLR2/MyD88-mediated Th2-biased immune response to primary DENV infection could favor secondary DENV infection to DHF/DSS *via* ADE (George et al., 2017). Therefore, balanced and durable immunity to all four DENV serotypes is of great importance for dengue vaccine development and avoiding the danger of ADE in subsequent infections. The lack of a suitable validated immunocompetent small animal model for vaccine testing and of defined immune correlates of protective immunity also represent a significant obstacle to vaccine development efforts (Idris et al., 2021). Therefore, it is important to investigate an alternative suitable immunocompetent animal model that could be used for testing vaccine efficacy (Jiang et al., 2021). Travel-associated DENV infection is a threat in dengue-endemic countries (Halstead and Wilder-Smith, 2019), and controlling travel-associated dengue vaccines for travelers is essential. Therefore, the dosages of vaccines also need to be considered during their development; for example, Dengvaxia requires three doses six months apart, which may limit its use in travelers. However, the TAK-003 and TV003/TV005, which are currently under phase III clinical trials and yet to obtain licenses, are currently being considered for two doses three months apart and a single dose, respectively. Moreover, the limited number of studies also hinders the identification of the underlying mechanism(s) of vaccine efficacy for developing a universal dengue virus. Clinical trials with a leading candidate vaccine demonstrated that unbalanced replication and immunodominance of one vaccine component over others may result in low efficacy and vaccine-induced severe disease (Nivarthi et al., 2021). Selection of attenuated DENV vaccine candidates based on plaque size led to mixed safety outcomes in clinical trials, which compromise the use of plaque size as an indicator of DENV attenuation (Bifani et al., 2021). Therefore, a reliable marker of DENV attenuation is important for vaccine candidates. Notably, a recent study indicated that genome diversity of DENV could be developed as a marker of DENV attenuation (Bifani et al., 2021). Another obstacle in dengue vaccine development is that virus-neutralizing antibodies do not invariably correlate with vaccine efficacy; therefore, other markers that may predict protection, including cell-mediated immunity, are urgently needed. A recent study showed the induction of DENV-specific CD4 + T cell responses after vaccination with a monovalent purified inactivated virus (PIV) vaccine candidate against DENV-1 adjuvanted with alum (Friberg et al., 2020). A better understanding of antibody responses that correlate with protection against DENV infection by all four serotypes is of great importance for developing a uniformly effective vaccine (Martinez et al., 2021). Recently, mRNA vaccines have shown the potential to be used against SARS-CoV-2 infection and successfully combatting the pandemic, although long-term safety has yet to be confirmed (Cai et al., 2021). A previous study also showed the potential of an mRNA vaccine encoding DENV1-NS-induced immunogenicity and protection in transgenic mice (Roth et al., 2019). Therefore, mRNA vaccines may open a new window for an effective DENV vaccine. Based on this information, it is important to enhance vaccine efficacy by improving the design of safe, effective, and affordable vaccines against dengue, including the use of various adjuvants such as TLR agonist adjuvants (Van Hoeven et al., 2018; Bidet et al., 2019).

The concept of trained immunity or innate immune memory is not only present in plants or invertebrates, but also in mammals (Quintin et al., 2014; Netea et al., 2016). TLRs are considered as the important triggerering molecule of trained immunity (Sanchez-Ramon et al., 2018), and have been dicussed in several recently published reviews (Netea et al., 2020;Owen et al., 2020;Pulendran et al., 2021). TLR agonists as vaccine adjuvants are currently under investigation for different human vaccines and appear as promising in the vaccine study (Bendelac and Medzhitov, 2002; Duthie et al., 2011; Pulendran et al., 2021). TLR agonist(s) may activate specific TLR(s) and enhance vaccine efficacy without direct participation in the protective immunity (Kumar et al., 2019). TLRs have been shown to activate Th1 response, and control and shape adaptive immune responses (Schnare et al., 2001). Effective TLR agonists enhance the targeted cellular or humoral adaptive responses that have been observed in several vaccines (Giannini et al., 2006; Del Giudice et al., 2018; Moser et al., 2020). There are several well-known TLR agonists, including triacylated lipopeptides [e.g., Pam3CysSerLys4 (Pam3CSK4) and their derivatives for TLR1/2, poly I:C (synthetic dsRNA) for TLR3, bacterial lipopolysaccharide or Monophosphoryl lipid A for TLR4, bacterial flagellin for TLR5, imiquimod and resiquimod (nucleoside analog) for TLR7/8, and CpG ODN for TLR9, which could be investigated for the utilization as dengue vaccine adjuvants (Duthie et al., 2011; Bidet et al., 2019)]. Although the understanding of TLR response to DENV infection made in this study should be an aid in the selection of TLR agonists to be used in dengue vaccine, however, an extensive research requires to find out the potential TLR agonist candidate for dengue vaccine. In addition, although vaccine containing TLR agonist should boost vaccine efficacy, the safety issues related to enhanced TLR signaling pathways due to TLR agonists require to be critically evaluated, which constitute an area of active investigation.

## Conclusion

The development of a safe and efficacious vaccine that can be used against all DENV serotypes, regardless of serostatus, remains a large challenge; however, its development may benefit from a better understanding of host innate immune responses, particularly interaction between TLRs and viral components. Several studies have shown the potential of TLR agonists as vaccine adjuvants, which could be investigated in the case of candidate DENV vaccines to enhance their protective efficacy. Furthermore, bridging and post-licensure studies are required to extend the conclusions concerning vaccine characteristics, and coadministration trials are necessary in pediatrics (Hombach, 2009). It is difficult to achieve effective, long-lasting, and uniform protection against all four DENV serotypes with the existing vaccine; it is also challenging to develop a safe and effective pan-serotype dengue vaccine. Therefore, the development of therapeutic agents against DENV and vector control programs should be enhanced. Until an effective vaccine is available, the mainstays of dengue prevention, such as disease surveillance and vector population control, should be properly implemented.

## Author Contributions

MEHK, MK, and KT-K: conceptualization and writing – review and editing. MEHK: writing – original draft preparation. KT-K: supervision. All authors have read and agreed to the published version of the manuscript.

## Conflict of Interest

The authors declare that the research was conducted in the absence of any commercial or financial relationships that could be construed as a potential conflict of interest.

## Publisher’s Note

All claims expressed in this article are solely those of the authors and do not necessarily represent those of their affiliated organizations, or those of the publisher, the editors and the reviewers. Any product that may be evaluated in this article, or claim that may be made by its manufacturer, is not guaranteed or endorsed by the publisher.
